# Atrial Septal Defect in an Elderly Woman–a Case Report

**Published:** 2011-02-25

**Authors:** CC Diaconu

**Affiliations:** ‘Carol Davila’ University of Medicine and Pharmacy, Bucharest, Ilfov Clinical Hospital, Internal Medicine Department, BucharestRomania

**Keywords:** echocardiography, transthoracic, malformation

## Abstract

**Rationale.** The atrial septal defect is one of the most common congenital anomalies in adults, but it is rarely diagnosed. It is characterized by a defect in the interatrial septum that allows pulmonary venous return to pass from the left to the right atrium.

**Objective.** A case of a 75–year–old female who presented with dyspnea, orthopnea, lower extremities swelling and palpitations is reported here. She had a 3 years history of atrial fibrillation and one–year history of cardiac failure NYHA (New York Heart Association) class Ⅱ.

**Methods and results.** Transthoracic echocardiography revealed a dilated right atrium of 74,2 mm, a dilated left atrium of 55,2 mm, a dilated left ventricle of 64/72,3 mm, a dilated right ventricle of 44,4 mm. Atrial septal defect ostium secundum type, with left–to–right shunt. Severe tricuspid insufficiency with a maximum gradient of 55,4 mm Hg. 4th degree mitral insufficiency. Severe pulmonary hypertension of 75 mm Hg. The ejection fraction of 29%. Atrial fibrillation. Interventricular sept with paradoxical motion.

**Discussion.** Ostium secundum defect is the most common type of atrial septal defect and accounts for 60–70% of all cases. The malformation often goes unnoticed for decades because symptoms may be absent and because physical signs are subtle. Symptoms usually take 30–40 years to develop. They are the consequences of pulmonary hypertension, atrial tachyarrhythmias and, sometimes, associated mitral valve disease.

The echocardiography can establish the size and location of the atrial septal defect, the magnitude and hemodynamic impact of the left–to–right shunt, and the presence and degree of pulmonary hypertension. The particularity of this case is that the patient lived for over 70 years almost asymptomatic.

## Introduction

The atrial septal defect is one of the most common congenital anomalies in adults, but it is rarely diagnosed. It is characterized by a defect in the interatrial septum that allows pulmonary venous return to pass from the left to the right atrium, resulting in right atrial and right ventricular chamber dilatation, the extent of which depends on the size of the shunt. The direction and size of the shunting are determined by the size of the defect and compliance of the ventricles. A small defect of less than 0,5 cm in diameter may be associated with a small shunt and no significant sequelae. A larger defect, of more than 2 cm in diameter, may be associated with a large shunt with important blood flow changes.  In most of the cases with atrial septal defects, the right ventricle is more flexible than the left; thus, the left atrial oxygenated blood is shunted to the right atrium, causing increased blood flow and enlargement of the right atrium, right ventricle and pulmonary arteries. If the right ventricle fails, the shunt may reverse and go from right to left [1,2,3]. 

## Meth

A 75–year–old female presented in the Emergency Department of Ilfov Clinical Hospital, Bucharest, Romania, with dyspnea, orthopnea, lower extremities swelling, palpitations. She had a 3 years history of atrial fibrillation diagnosed by her family doctor and one-year history of cardiac failure NYHA (New York Heart Association) class Ⅱ. 

Physical examination revealed an irregular pulse 80 beats/min, blood pressure of 100/60 mmHg, bi–basal crackles on chest auscultation, lower extremities swelling. Heart sounds examination revealed a IV^th^ degree pansystolic murmur at the left border of the stern. The jugular veins were dilated. The patient complained of pain in the upper right abdominal region, enhanced by palpation. The liver margin was tender, round, of 6 cm below the costal rib, with a smooth liver surface. The spleen could not be felt. 

EKG: atrial fibrillation of 80/min, right bundle branch block, infero-lateral ischemia.

Transabdominal ultrasound showed a homogenous, enlarged liver. The suprahepatic veins were dilated, as well as the inferior vena cava (30 mm, without respiratory variations). 

## Results

**Figure 1:**
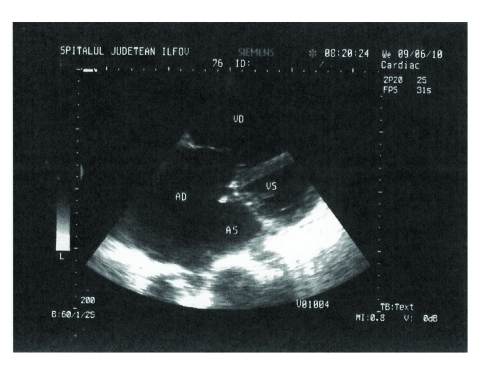
2D Transthoracic echocardiography. The right atrium dilated in apical 4 chamber–view. AD=right atrium, AS=left atrium, VS=left ventricle, VD=right ventricle.

**Figure 2:**
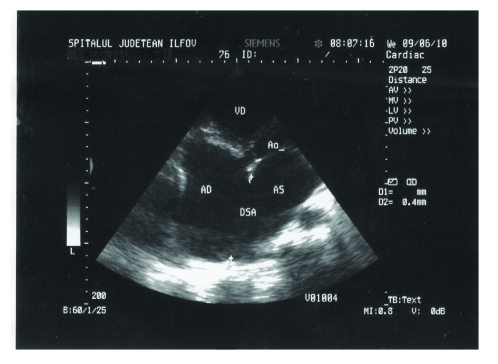
2D Transthoracic echocardiography. Atrial septal defect in apical 4 chamber–view. AD=right atrium, AS=left atrium, VD=right ventricle, A0=aorta, DSA=atrial septal defect.

**Figure 3:**
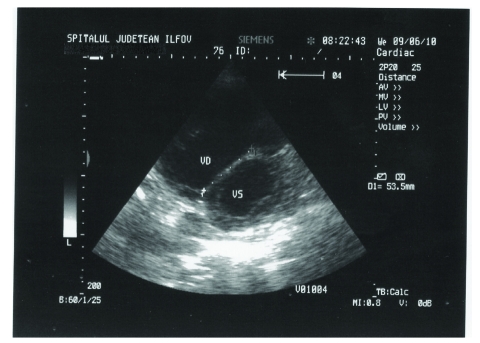
2D Transthoracic echocardiography. The flattening of the interventricular septum in parasternal short–axis view. VS=left ventricle, VD=right ventricle.

Transthoracic echocardiography revealed a dilated right atrium of 74,2 mm (Figure 1), dilated left atrium of 55,2 mm, dilated left ventricle of 64/72,3 mm, dilated right ventricle of 44,4 mm. Atrial septal defect ostium secundum type, of 6 cm (Figure 2), with left–to–right shunt. Severe tricuspid insufficiency with maximum gradient of 55,4 mm Hg. 4^th^ degree mitral insufficiency. Severe pulmonary hypertension of 75 mm Hg. The ejection fraction of 29%. Atrial fibrillation. Interventricular sept with paradoxical motion. Flattening of the interventricular septum (Figure 3). 

The diagnosis was IVth degree chronic heart failure NYHA. Atrial septal defect ostium secundum type with left–to–right shunt. Severe tricuspid insufficiency. 4th degree mitral insufficiency. Severe pulmonary hypertension of 75 mm Hg. Chronic atrial fibrillation. Right bundle branch block. 

## Discussion

Atrial septal defect accounts for 10% of all congenital heart diseases and for 22–40% of congenital heart diseases in adults. Ostium secundum defect is the most common type and accounts for 60–70% of all cases.  Atrial septal defect occurs with a female/male ratio of approximately 2:1. Patients with ASD are usually asymptomatic in infancy and childhood. Symptoms appear with aging. By the age of 40 years old, 90% of untreated patients have symptoms of exertional dyspnea, fatigue, palpitation or sustained arrhytmia [4]. Our patient had only a three–year history of atrial fibrillation, despite her age of 75 years.

In atrial septal defect, the malformation of the atrial septum may occur in various positions:

- In the lower part–ostium primum, 15% of the cases.
- Ostium secundum, in area of fossa ovalis, 75%.
- Upper atrial septum, sinus venosus, 10% of the cases.

The malformation often goes unnoticed for decades because symptoms may be absent and because physical signs are subtle. Most children are asymptomatic, though some may present easy fatigability and exertional dyspnea. Symptoms usually take 30–40 years to develop. They are the consequences of pulmonary hypertension, atrial tachyarrhythmias and, sometimes, associated mitral valve disease. The particularity of this case is that the patient lived for over 70 years almost asymptomatic.

The echocardiography can establish the size and location of the ASD, the magnitude and hemodynamic impact of the left–to–right shunt, and the presence and degree of pulmonary hypertension [5,6,7,8]. 

The decision to repair any kind of ASD is based on clinical and echocardiographic information, including the size and location of the ASD, the magnitude and hemodynamic impact of the left-to-right shunt, and the presence and degree of pulmonary hypertension [9].

Long–term prevention of death and complications is best achieved when the ASD is closed before the age of 25 years and when systolic pressure in the main pulmonary artery is less than 40 mm Hg [9].

Although life expectancy is not normal, patients generally survive into adulthood without surgical intervention and many patients live to advanced age. Advanced pulmonary hypertension seldom occurs before the 3^rd^ decade. Late complications are stroke and atrial fibrillation.
